# Supplementary material for: Emotional Effects in Object Recognition by the Visually Impaired People in Grocery Shopping
Source: Sensors (Basel). 2022 Nov 3;22(21):8442. doi: 10.3390/s22218442 (PMC9654971; doi:10.3390/s22218442)
Supplement: Supplementary file 1 [file sensors-22-08442-s001.zip › sensors-1933854-supplementary.pdf]

Table S1. Summary details of behavioral, self-report, and autonomic data for visually impaired and control group (Mean ± Standard Deviation)

		**Visually impaired group**	**Control group**
Behavioral data	ACC fruit	1.89±0.33	2.00±0.00
	ACC pasta	1.22±0.67	2.00±0.00
	ACC frozen food	1.44±0.53	2.00±0.00
	RTs fruit	24.76±7.90	28.96±9.99
	RTs pasta	27.27±7.98	19.43±7.30
	RTs frozen food	27.89±6.07	19.56±7.44
Self-report data	Perceived stress level	3.00±1.58	2.50±1.38
	Disorientation	3.11±1.69	1.42±0.67
	Enjoyable experience level	5.33±2.29	4.92±1.38
	Simplicity in finding products	3.33±1.00	4.75±0.62
	Self confidence in repeating the route independently	2.78±1.30	4.92±0.29
Autonomic data	SCL fruit	1.87±1.95	8.36±12.61
	SCL pasta	2.09±2.44	9.28±12.25
	SCL frozen food	2.17±2.98	8.33±10.49
	SCR fruit	0.01±0.02	-0.02±0.11
	SCR pasta	0.01±0.04	0.02±0.05
	SCR frozen food	-0.04±0.10	-0.03±0.10
	BVP fruit	49.91±0.34	49.81±0.35
	BVP pasta	49.43±0.72	49.99±0.42
	BVP frozen food	49.79±0.76	49.97±0.59
	PVA fruit	26.36±22.18	15.16±8.86
	PVA pasta	39.52±15.49	14.98±11.58
	PVA frozen food	32.94±17.16	10.94±4.13
	PULS fruit	69.49±12.72	75.10±12.67
	PULS pasta	69.88±11.54	77.56±16.91
	PULS frozen food	70.86±11.94	75.22±8.12

Abbreviations: ACC, Accuracy; RTs, Reaction Times; SCL, Skin Conductance Level; SCR, Skin Conduct Response; BVP, Blood Volume Pulse; PVA, Pulse Volume Amplitude
